# HIV Treatment-As-Prevention Research: Taking the Right Road at the Crossroads

**DOI:** 10.1371/journal.pmed.1001800

**Published:** 2015-03-10

**Authors:** Richard Hayes, Sarah Fidler, Anne Cori, Christophe Fraser, Sian Floyd, Helen Ayles, Nulda Beyers, Wafaa El-Sadr, HPTN 071 (PopART) Study Team

**Affiliations:** 1 London School of Hygiene & Tropical Medicine, Department of Infectious Disease Epidemiology, London, United Kingdom; 2 Imperial College London, Department of Medicine, London, United Kingdom; 3 Imperial College London, Department of Infectious Disease Epidemiology, London, United Kingdom; 4 ZAMBART, University of Zambia, School of Medicine, Lusaka, Zambia; 5 London School of Hygiene & Tropical Medicine, Department of Clinical Research, London, United Kingdom; 6 Desmond Tutu TB Centre, University of Stellenbosch, Department of Paediatrics and Child Health, Stellenbosch, South Africa; 7 Columbia University, Mailman School of Public Health, New York, New York, United States of America

## Abstract

Reflecting on a Policy Forum article by Till Bärnighausen and colleagues, the HPTN 071 (PopART) Study Team consider ethical and study power concerns and the importance of the trial’s future findings.

## Trials of Treatment-As-Prevention

Despite recent reductions in HIV incidence in several countries in sub-Saharan Africa, incidence still remains at unacceptably high levels [1]. Effective control of the epidemic requires more intensive prevention efforts. Two important approaches to address this goal are “combination prevention” [2], in which a number of partially effective interventions are combined to achieve a substantial reduction in HIV incidence, and “treatment-as-prevention” (TasP), offering antiretroviral therapy (ART) to all HIV-infected adults irrespective of CD4+ T-cell count to prevent onward transmission of HIV [3].

Mathematical models of the effects of TasP on population-level HIV incidence have produced a wide range of projections [4–7]. There remain many uncertainties in model assumptions and, critically, the impact of TasP at a population level depends on the coverage and uptake of HIV-testing, linkage to care, treatment initiation and adherence—the “cascade of care” [8]. Rigorous empirical studies are needed to determine whether TasP programmes can be implemented successfully in practice; measure these important process indicators; assess the balance between harms, costs, and benefits; and evaluate the impact of TasP on HIV incidence at population level.

Four large community trials are currently underway in South Africa [9,10], Zambia [10], Botswana [11], and Kenya and Uganda [12]. The trials are studying a range of intervention strategies with important differences in study design, but all four are measuring the impact of TasP on HIV incidence using a community-randomised design.

In June 2014, Till Bärnighausen and colleagues [13] presented their views on the implications of the 2013 change in WHO ART guidelines for the TasP studies. Their main conclusions were that as WHO guidelines are implemented [14], it will become unethical to continue the trials because the new guidelines cannot be withheld in control communities; that if the new guidelines are adopted in the control communities, the trials will no longer be adequately powered; and that alternative approaches such as pooling of data or adoption of stepped-wedge study designs should be considered.

**Fig 1:**
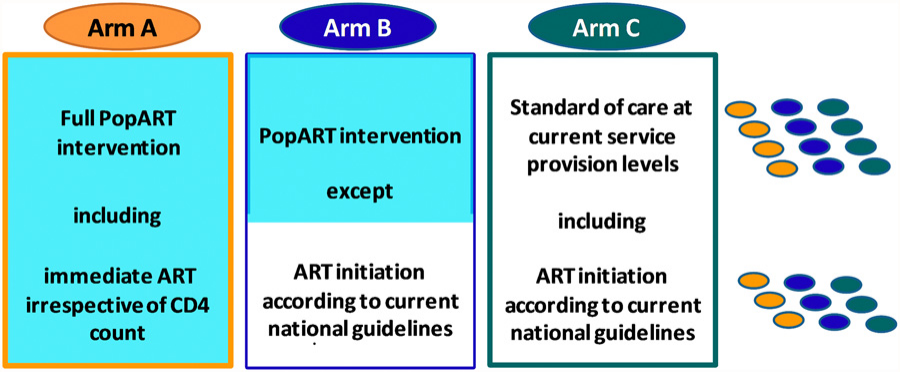
Design of HPTN 071 (PopART) study showing the three study arms. This is a three-arm cluster-randomised trial with 21 communities (*n* ≈ 1.2 million total population). The three matched triplets (three in South Africa, four in Zambia) are illustrated on the right of the diagram, with one community per study arm in each triplet.

We believe that the article by Bärnighausen and colleagues contains a number of inaccurate statements that compromise their conclusions. We discuss these issues in relation to the HPTN 071 (PopART) trial that our study team is carrying out in 21 communities in Zambia and South Africa to measure the impact of a combination prevention package, including universal HIV testing and treatment, on population-level HIV incidence [10]. The HPTN 071 (PopART) trial has three study arms (Fig. 1).

The communities in Arm A will receive the full “PopART” combination prevention programme, including the offer of immediate initiation of ART for all HIV-infected adults irrespective of CD4 count. The PopART package includes home-based provision of HIV testing and counselling, for all household members, referral of HIV-negative men for voluntary male medical circumcision, screening for symptoms of tuberculosis and sexually transmitted infections, and referral of all HIV-infected individuals to the local health facility with active linkage, follow-up, and ART adherence support. Arm B communities will receive the full PopART package except that ART will be provided according to the prevailing local treatment guidelines. Arm C communities will continue to receive current standard of care.

## Ethics of Trial Continuation

Bärnighausen and colleagues imply that when the study countries adopt the 2013 WHO treatment guidelines, the TasP trial protocols would require the control communities to continue receiving ART according to previous guidelines, with ART initiated at a CD4+ count of <350 cells/mm^3^. However, this assumption does not apply to HPTN 071 (PopART). The HPTN 071 trial protocol [10] states that any changes in treatment guidelines during the course of the trial will be implemented in Arms B and C. Zambia has already adopted and is introducing the 2013 WHO guidelines into communities in Arms B and C, and South Africa is now following.

There is therefore no ethical concern with the continuation of the HPTN 071 trial with adoption of the new WHO guidelines.

## Inadequate Study Power

Bärnighausen and colleagues state that if the control arms of the TasP trials do switch to providing ART according to the 2013 WHO guidelines, the studies will be underpowered for answering the primary study question, because of much smaller differences between the study arms.

With respect to HPTN 071 (PopART), this is incorrect. The impact of HIV treatment on transmission at a population level critically depends not only on the eligibility criteria for ART initiation but also on several other key variables, including the uptake and coverage of HIV testing, linkage to care, treatment initiation, and adherence. At each step in this cascade of care there are substantial challenges in achieving high coverage and, without effective measures to increase service uptake, the impact of any treatment programme (and of changes in ART eligibility) will be limited.

The design of HPTN 071 (PopART) was informed by the results of mathematical modelling of the projected impact of the interventions in Arms A and B under a range of assumptions about uptake and coverage of the various components of the intervention [10,15]. At the request of the study’s Data and Safety Monitoring Board (DSMB), the model was used to evaluate the effects on study power if the study countries were to adopt the 2013 WHO guidelines (S1 Text). In brief, the projections show only a small reduction in impact when Arm A is compared with Arm C. This is because the effects of the change in eligibility criteria in Arm C will have only a limited effect without substantial increases in uptake and coverage of testing and linkage. The study power for the Arm A versus C comparison, the main study comparison, remains very high. The difference between Arms B and C will increase following adoption of the new guidelines, leading to higher power for this comparison. This is because more patients will be eligible for treatment under the 2013 guidelines, but uptake and coverage will be higher in Arm B due to the PopART home-based services. Finally, the difference between Arms A and B will decrease. Both of these study arms benefit from the increased uptake and coverage achieved by the PopART home-based services. Following adoption of the 2013 guidelines, there will be a smaller number of HIV-infected individuals offered treatment in Arm A who would not be eligible for treatment if in Arm B communities, reducing the power to demonstrate a difference between Arms A and B.

## Importance of Implementation Science to Determine Effectiveness and Inform Policy on TasP

We agree with Bärnighausen and colleagues that an important objective of the TasP trials, in addition to measuring effects on HIV incidence, is to provide useful data on the implementation of TasP to guide future policy and practice. TasP will only reach its potential effect on HIV transmission if the uptake and coverage of HIV services is substantially expanded. Changing treatment guidelines alone is not sufficient to assure the population level benefits of TasP. For optimal impact, a large proportion of the population needs to know their HIV status (through regular testing and re-testing), with effective linkage to appropriate treatment and care. We therefore prefer the term “Universal Testing and Treatment” (UTT), which emphasises the importance of the entire cascade of care, and not just treatment provision [16]. There remains an urgent need for implementation science to provide information on how such HIV services can most effectively be delivered in resource-poor settings, if TasP is found to be effective in reducing population level HIV incidence.

HPTN 071 (PopART) will provide valuable data on a wide range of process indicators. Social science research will investigate the acceptability of the intervention to local communities, and case-control studies will explore factors related to uptake of the different steps of the cascade with detailed costing exercises to determine overall cost-effectiveness. It cannot be assumed that TasP carries no risks; the study will also measure behavioural risk disinhibition, ART toxicity, stigma, and drug resistance and balance these against effects on HIV incidence.

## Alternative Approaches

In their article, Bärnighausen and colleagues propose two alternative approaches to take forward the evaluation of TasP interventions.

They suggest first that it may be possible to pool data from the TasP trials to gain a more reliable overall measure of impact. This will be difficult, in practice, because of the substantial differences in the interventions being tested and the study designs, as well as differences in HIV transmission dynamics in different populations. We agree, however, that it will be important to bring together the data from the four studies in careful analyses, supported by mathematical modelling, to learn what we can from the findings. The investigators of the TasP trials have resolved to collaborate closely, facilitating future joint analyses.

Their second proposal is that if the trials cannot proceed to planned completion, it may be possible for the researchers to work with Ministries of Health to agree on a phased introduction of the new treatment guidelines according to a randomised stepped-wedge design. While this is an interesting proposal, it is unlikely that countries would be willing to delay initiation of new guidelines long enough for such a strategy to be feasible.

## Conclusions

In summary, we believe that the conclusions of Bärnighausen and colleagues are based on misunderstandings about the design of the HPTN 071 (PopART) trial. Because this trial is committed to providing care and treatment in Arms B and C according to the prevailing national guidelines, there are no ethical concerns with the continuation of the trial. We also show that the study, with its three-arm design, will remain highly powered for its main comparisons even with adoption of the 2013 WHO guidelines in the study countries. We do not consider the implications for other ongoing TasP trials in this article, but there may be value in exploring the effects of changing guidelines on the power of those studies. Ultimately, there is an urgent need to demonstrate effectiveness of the UTT approach at a population level and to rigorously evaluate how best to safely and effectively deliver such an approach, which can then inform international policy decisions.

## Supporting Information

S1 Text Mathematical model projections of impact of the HPTN 071 (PopART) trial interventions under the 2013 treatment guidelines. Note: This summary of the projections, prepared for the trial’s Data and Safety Monitoring Board, is provided for information and has not been subjected to journal peer-review.
(PDF)

## Abbreviations

ART: antiretroviral therapy
DSMB: Data and Safety Monitoring Board
TasP: treatment-as-prevention
UTT: universal testing and treatment
