# Supplementary material for: HIV Treatment-As-Prevention Research: Taking the Right Road at the Crossroads
Source: PLoS Med. 2015 Mar 10;12(3):e1001800. doi: 10.1371/journal.pmed.1001800 (PMC4355412; doi:10.1371/journal.pmed.1001800)
Supplement: S1 Text — Note: This summary of the projections, prepared for the trial’s Data and Safety Monitoring Board, is provided for information and has not been subjected to journal peer-review. (PDF) [file pmed.1001800.s001.pdf]

Potential impact of the new WHO guidelines on the power in the HPTN071-PopART trial - Mathematical modelling as submitted to the HPTN 071 (PopART) DSMB not peer-reviewed by PLOS Medicine

Executive summary

In June 2013, the WHO released new guidelines on the use of antiretroviral therapy (ART) for treating and preventing HIV infection, recommending broadening the spectrum of individuals eligible for initiation of ART [1]. The adoption by Zambia and South Africa of these new WHO guidelines would affect the HPTN-071 (popART) trial, in which individuals in arm C (standard of care) and B (universal testing but treatment according to national guidelines) are treated according to the national guidelines.

We extended the mathematical model developed to assist the trial design in order to assess the potential impact of the changes in national guidelines on the power of the trial to detect differences in 3-year cumulative HIV incidence between the different arms. The model is an extension of that described in a manuscript submitted for publication in PLoS one which is attached to the current document. New findings are presented in the same format for comparison.

We explored 5 scenarios in which the date of adoption of the new guidelines was varied as well as the proportion of the HIV positive individuals who would become eligible according to the new guidelines. If targets are reached, our model predicts that the HIV incidence over 3 years would be reduced by 61% to 56% in Zambia and 62% to 58% in South Africa in arm A and 25% to 37% in Zambia and 26% to 39% in South Africa in arm B, compared to arm C, depending on assumptions about the time of adoption the new guidelines and the expected size of the population affected by them. The earlier the new guidelines are adopted, and the more HIV positive individuals they affect, the larger the power of detecting a difference in HIV incidence over 3 years between arms B and C, but the smaller the power to detect a difference between arms A and B or A and C. However, even in the most extreme scenario, the power of detecting a difference between arms A and C would remain above 99%, and the power of detecting a difference between arms A and B would remain above 60%, the power of detecting a difference between arms B and C increasing to at least 75%.

Importantly, we found that the variability in the predicted reduction in HIV incidence in the intervention arms associated with uncertainty in the trial uptake and potential changes in behaviors at the community level was much larger than that associated with uncertainty in the date of adoption and impact of the new guidelines in country.

In summary, our model indicates that although the adoption of the new WHO guidelines in country will affect the reduction in HIV incidence in the intervention arms, this should not hamper our ability to detect differences in 3-year cumulative HIV incidence between the different trial arms.

Context

In June 2013, WHO released new guidelines on the use of antiretroviral therapy (ART) for treating and preventing HIV infection [1]. Based on recent evidence that early ART initiation helps infected individuals to live a longer and healthier life and prevents transmission of the virus to others, WHO recommends broadening the spectrum of individuals eligible for ART. The new guidelines recommend initiating treatment in

- HIV+ adults with CD4<500 cells/mm3,
- HIV+ individuals in a serodiscordant couple,
- HIV+ individuals with active TB or hepatitis B,
- HIV+ pregnant and breastfeeding women,
- HIV+ children under 5 years of age.

The adoption by Zambia and South Africa of these new WHO guidelines is going to affect the HPTN-071 (popART) trial, in which individuals in arm C (standard of care) and B (universal testing but treatment according to national guidelines) are treated according to the national guidelines. Broadening the spectrum of eligible individuals is likely to increase the relative difference in 3-year cumulative incidence between arms B and C but to decrease the relative difference between arms A and B. The aim of this document is to use mathematical modeling to project the impact of the trial on 3-year cumulative incidence under different scenarios of adoption of the new guidelines in the two countries.

Methods

The basic model is the one described in [2]. Here we consider 5 variations of this model to account for different scenarios of adoption of the new WHO guidelines in country, which vary in terms of time of adoption and proportion of the population affected by the new guidelines. The scenarios considered are summarized in Table 1, and described in more detail in appendix 1.

Table 1: Scenarios considered

Original paper	▪ Trial starts on 1st July 2013<br>▪ National guidelines don't change<br>(see [2])
No changes in guidelines	▪ Trial starts on 1st November 2013<br>▪ National guidelines don't change
Late adoption – small eligibility	▪ Trial starts on 1st November 2013<br>▪ National guidelines change on 1st January 2015<br>▪ 40 % of pregnant women attend antenatal clinic and undertake HIV testing and linkage to care<br>▪ 5% of the HIV+ individuals with CD4>500 are in a serodiscordant couple or co-infected with TB or Hepatitis B
Late adoption – large eligibility	▪ Trial starts on 1st November 2013<br>▪ National guidelines change on 1st January 2015<br>▪ 90 % of pregnant women attend antenatal clinic and undertake HIV testing and linkage to care<br>▪ 30% of the HIV+ individuals with CD4>500 are in a serodiscordant couple or co-infected with TB or Hepatitis B

Early adoption – small eligibility	▪ Trial starts on 1st November 2013<br>▪ National guidelines change on 1st January 2014<br>▪ 40 % of pregnant women attend antenatal clinic and undertake HIV testing and linkage to care<br>▪ 5% of the HIV+ individuals with CD4>500 are in a serodiscordant couple or co-infected with TB or Hepatitis B
Early adoption – large eligibility	▪ Trial starts on 1st November 2013<br>▪ National guidelines change on 1st January 2014<br>▪ 90 % of pregnant women attend antenatal clinic and undertake HIV testing and linkage to care<br>▪ 30% of the HIV+ individuals with CD4>500 are in a serodiscordant couple or co-infected with TB or Hepatitis B

Results

Here we briefly present the predicted HIV incidence in arm C as well as incidence reduction in arms A and B under the 5 scenarios considered regarding adoption of WHO guidelines in country.
Table 2 presents results for 3-year cumulative incidence.
Figures 1 to 6 present the dynamic of prevalence and incidence over time (equivalent of Figure 3 in [2]).
Figures 7 to 12 show the relative reduction in 3-year cumulative HIV incidence in arms A and B when parameters relating to the uptake of the interventions are varied (Figure 4 in [2]).
Figures 13 to 18 show the relative reduction in HIV incidence in arms A and B under 10 parameter sets calibrated to the UNAIDS prevalence estimates (Figure S6 in [2]).

As expected, the adoption of WHO guidelines in country increases the difference in predicted HIV incidence between arms B and C, and decreases that between arms A and C. Under the central target, the relative reduction in 3-year cumulative incidence in arm A ranged from 61% to 56% in Zambia and 62% to 58% in South Africa, depending on assumptions about the time of adoption and expected impact of the new guidelines (see Table 2). In arm B, it ranged from 25% to 37% in Zambia and 26% to 39% in South Africa (see Table 2). The adoption of the new guidelines would therefore increase our power to detect a difference between arms B and C. Moreover, in all scenarios considered, the power of detecting a difference between arms A and C, although reduced by the adoption of the new guidelines, was above 99%. The adoption of the new guidelines would decrease our power to detect differences between arms A and B, but under the most extreme scenario (early adoption of the new guidelines and large eligibility), we estimated that the power for detecting a difference between arms A and B would remain above 60% (see the HPTN071-PopART protocol and appendix 2 for detail on power calculations). In this scenario, the power of detecting a difference between arms B and C would increase to at least 75%.

Interestingly, the variability in the predicted reduction in HIV incidence in the intervention arms associated with uncertainty in the trial uptake and potential changes in behaviors at the community level was much larger than that associated with uncertainty in the date of adoption and impact of the new guidelines in country.

Table 1: Projected impact of the intervention on HIV incidence in Arms A and B compared with Arm C for central target under different scenarios regarding the adoption of WHO guidelines.

<table>
<tr><th rowspan="3">Scenario</th><th colspan="3">Zambia</th><th colspan="3">South Africa</th></tr>
<tr><th colspan="2">Relative reduction in 3-year cumulative incidence</th><th rowspan="2">Mean annual incidence rate in arm C over 3 years</th><th colspan="2">Relative reduction in 3-year cumulative incidence</th><th rowspan="2">Mean annual incidence rate in arm C over 3 years</th></tr>
<tr><th>Arm A</th><th>Arm B</th><th>Arm A</th><th>Arm B</th></tr>
<tr><td>Original paper (no change in guidelines, trial starts 1st July)</td><td>61%</td><td>25%</td><td>1.86%</td><td>63%</td><td>27%</td><td>1.37%</td></tr>
<tr><td>No change in guidelines, trial starts 1st November)</td><td>61%</td><td>25%</td><td>1.85%</td><td>62%</td><td>26%</td><td>1.36%</td></tr>
<tr><td>Late adoption of guidelines, small eligibility (see main text)</td><td>59%</td><td>31%</td><td>1.75%</td><td>61%</td><td>32%</td><td>1. 32%</td></tr>
<tr><td>Late adoption of guidelines, large eligibility (see main text)</td><td>58%</td><td>32%</td><td>1.68%</td><td>60%</td><td>33%</td><td>1.28%</td></tr>
<tr><td>Early adoption of guidelines, small eligibility (see main text)</td><td>58%</td><td>35%</td><td>1.67%</td><td>60%</td><td>37%</td><td>1.28%</td></tr>
<tr><td>Early adoption of guidelines, large eligibility (see main text)</td><td>56%</td><td>37%</td><td>1.53%</td><td>58%</td><td>39%</td><td>1.20%</td></tr>
</table>

Figure 1-6: Model fit and projections under central target scenario for Zambia (top row) and South Africa (bottom row). Left panels show HIV prevalence and right panels show annualized HIV incidence over time. The red, blue and black lines correspond to arms A, B and C respectively. The grey dots and error bars are the UNAIDS HIV prevalence estimates.

Figure 1: Original paper (no change in guidelines, trial starts 1st July 2013)

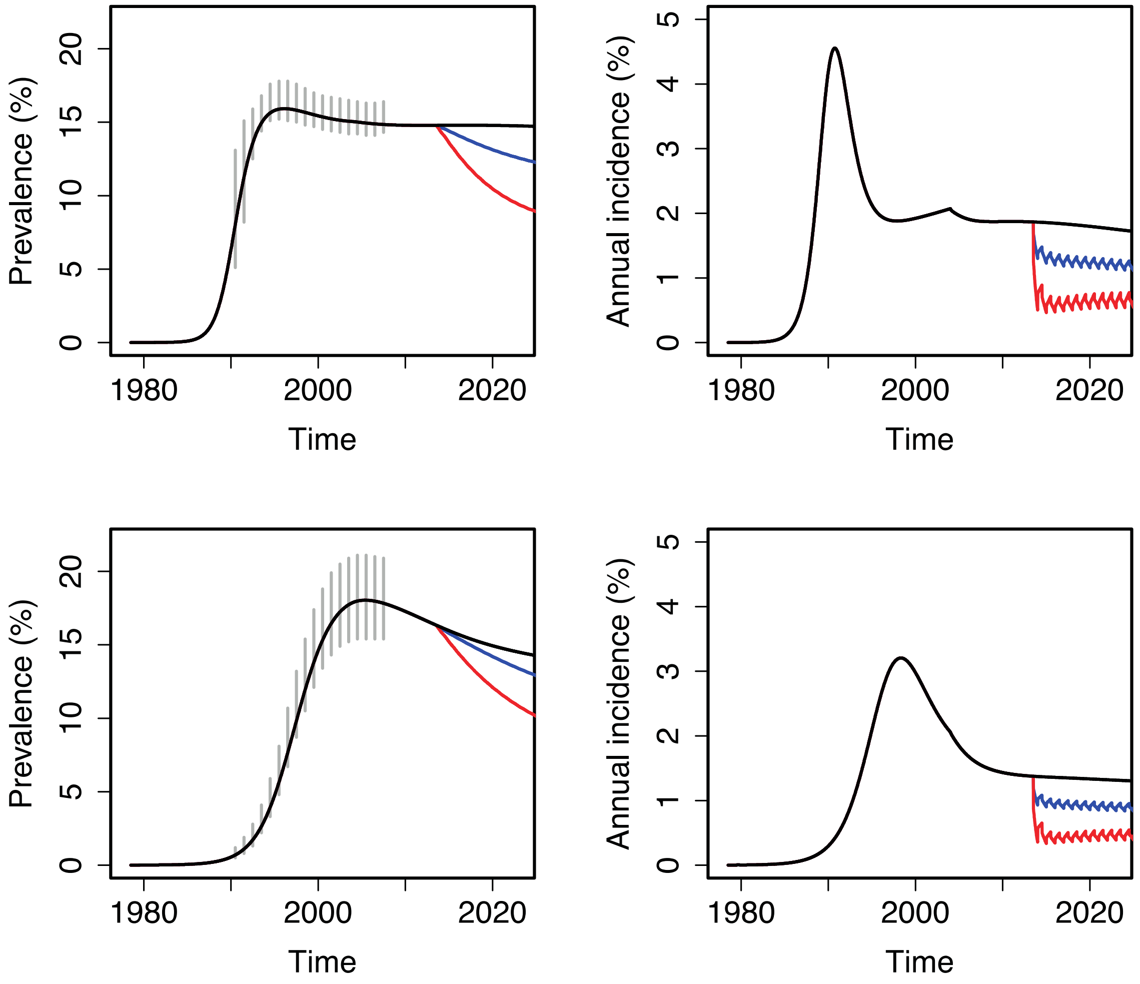

Figure 2: No change in guidelines, trial starts on 1st November 2013

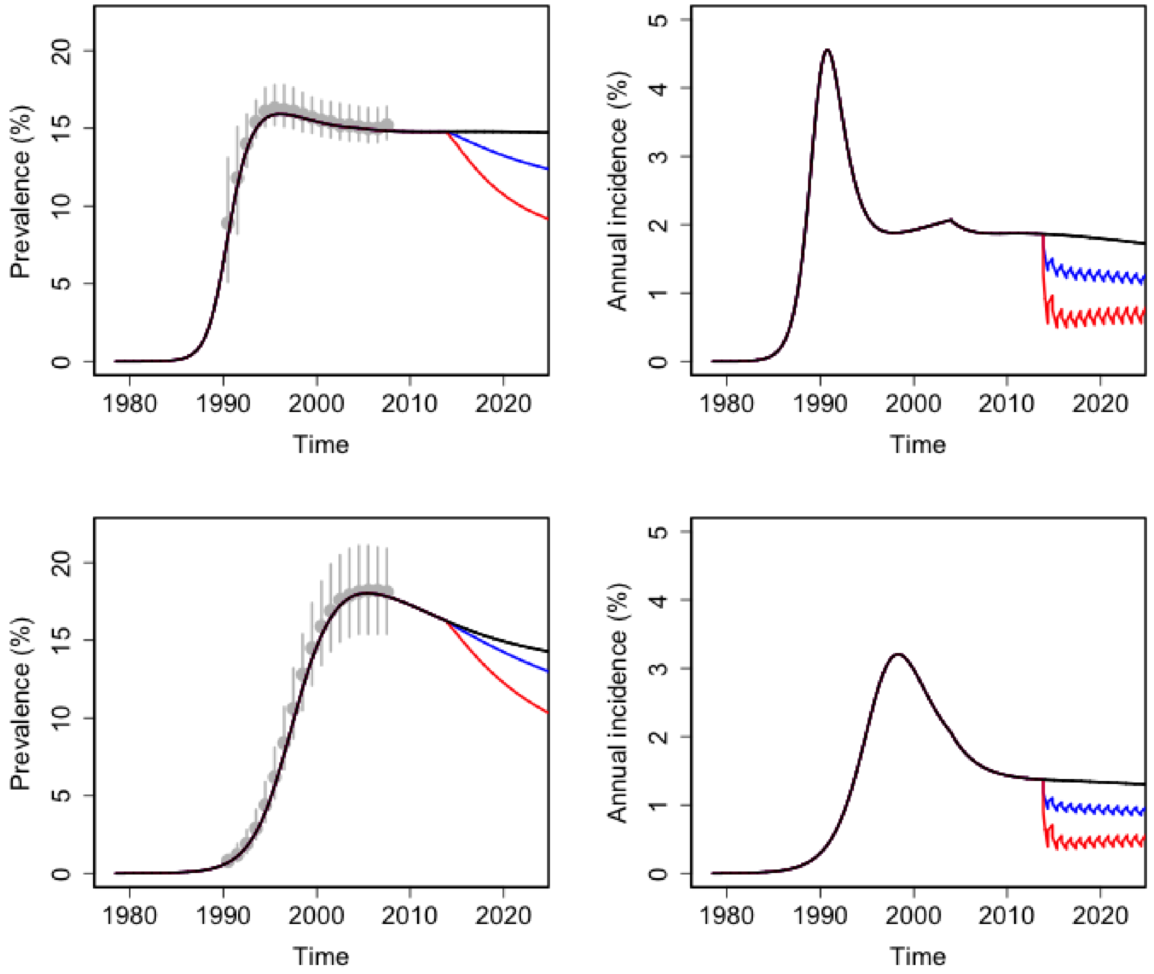

Figure 3: Late adoption of guidelines, small eligibility (see main text)

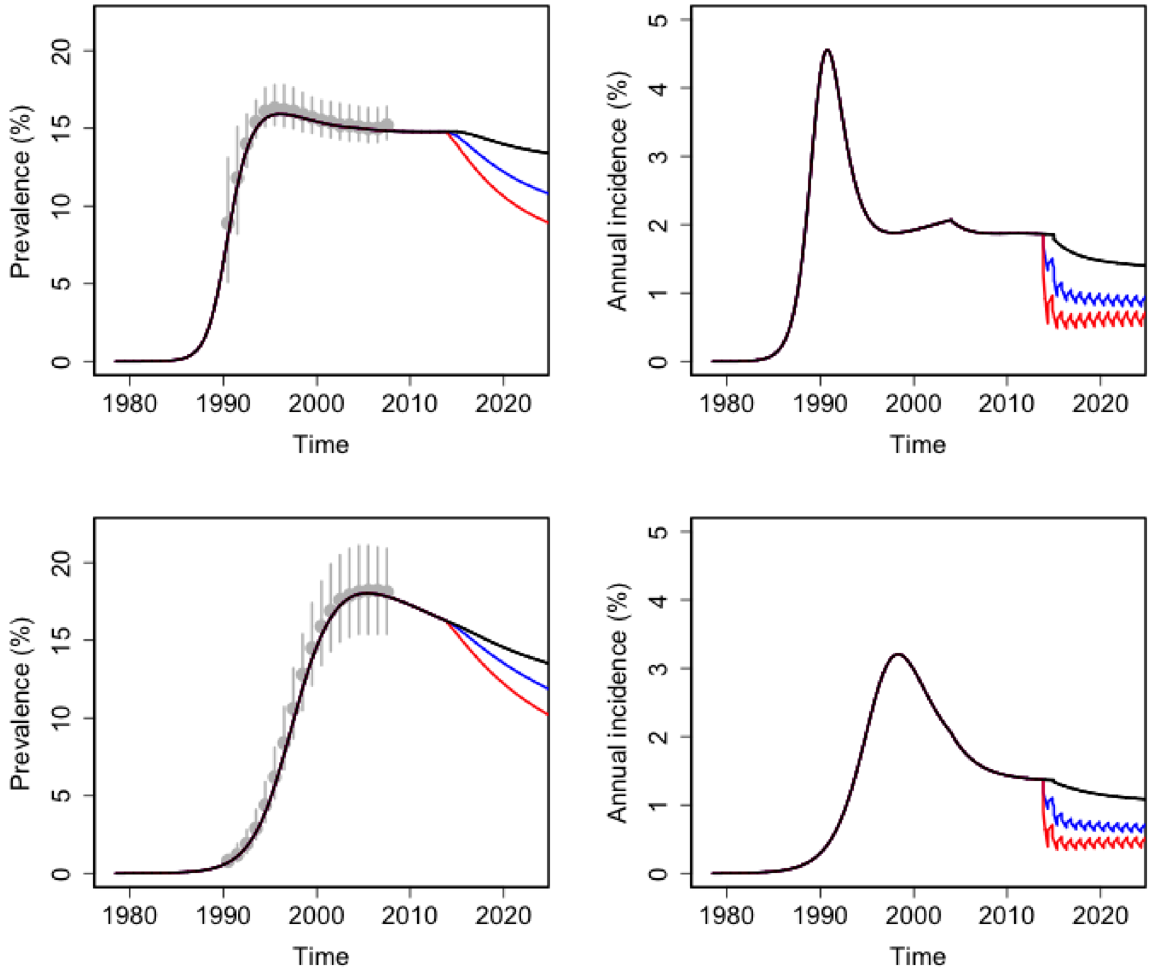

Figure 4: Late adoption of guidelines, large eligibility (see main text)

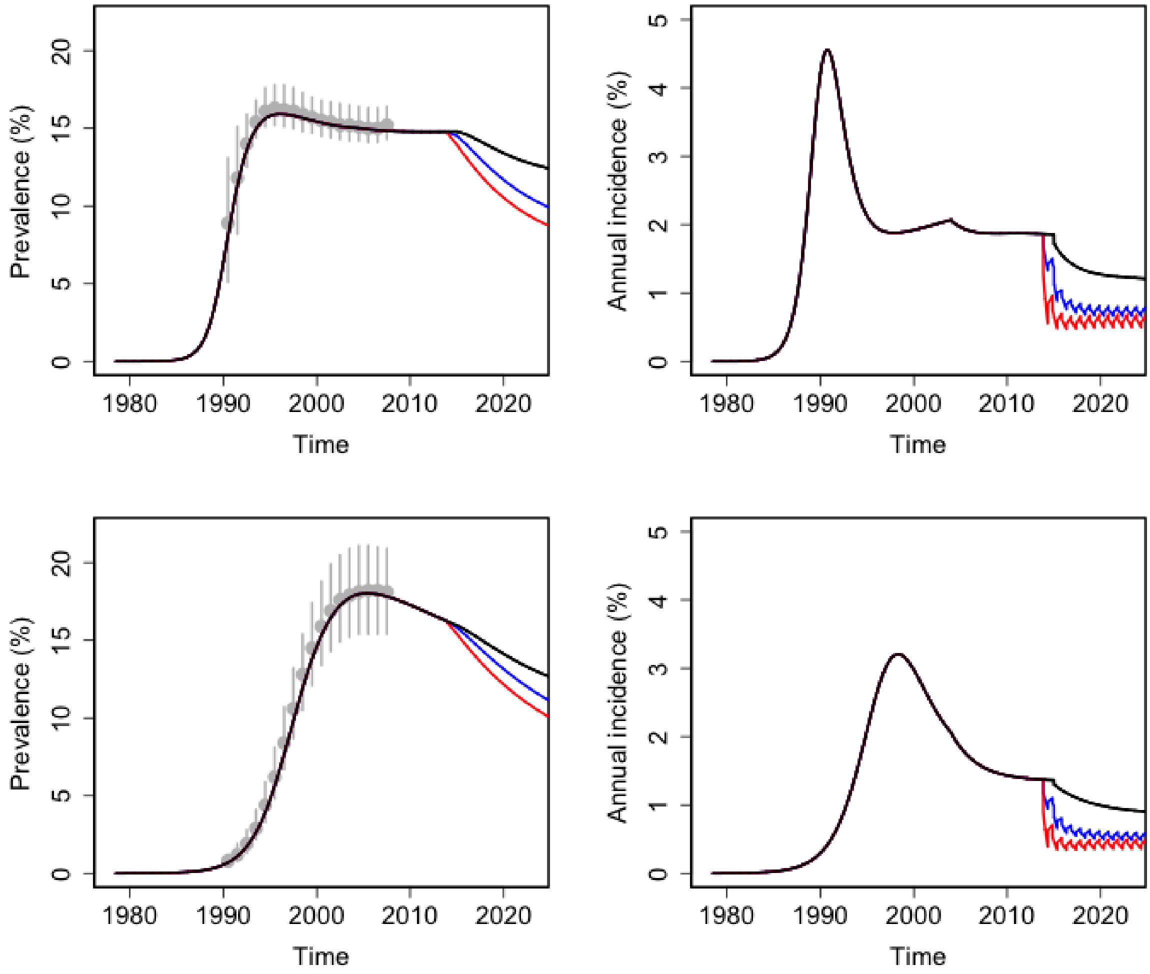

Figure 5: Early adoption of guidelines, small eligibility (see main text)

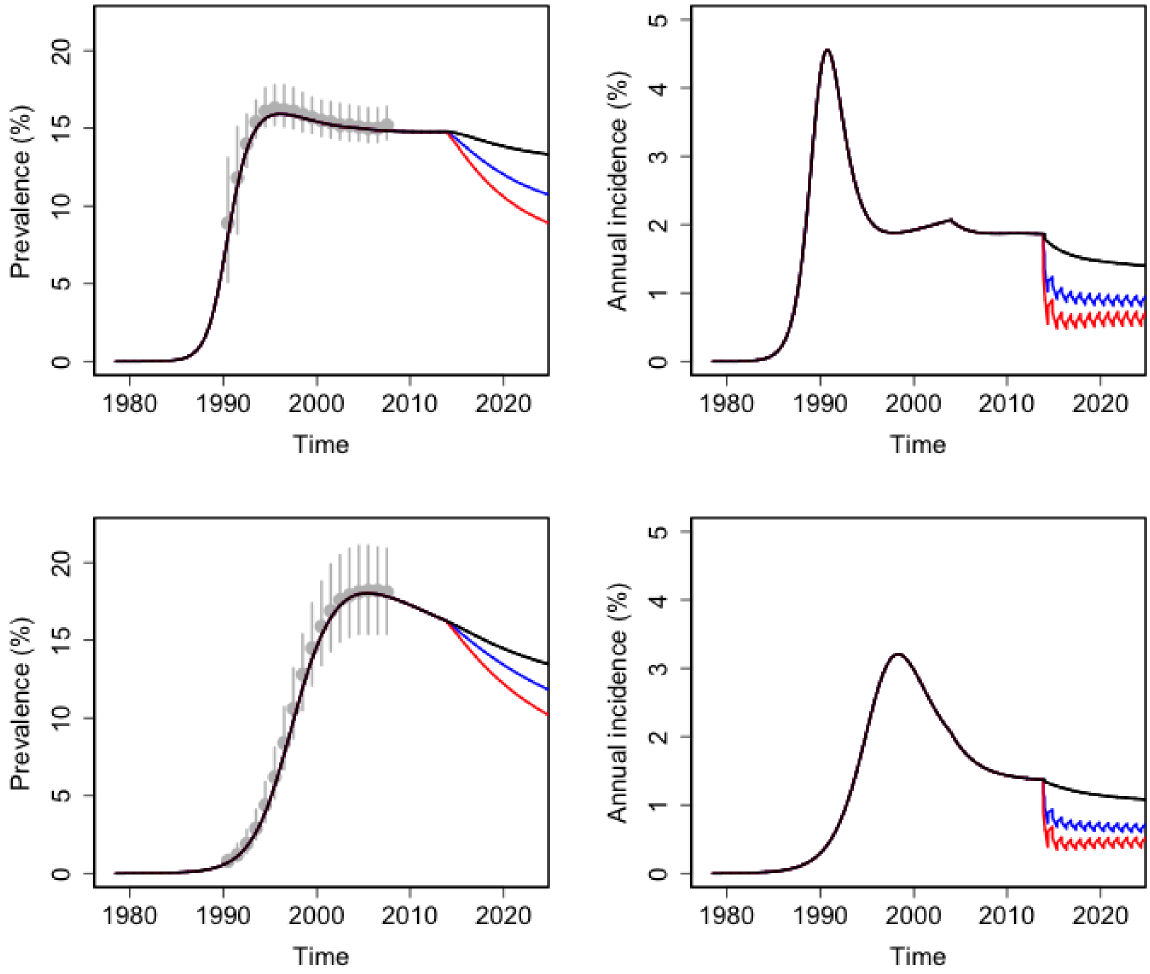

Figure 6: Early adoption of guidelines, large eligibility (see main text)

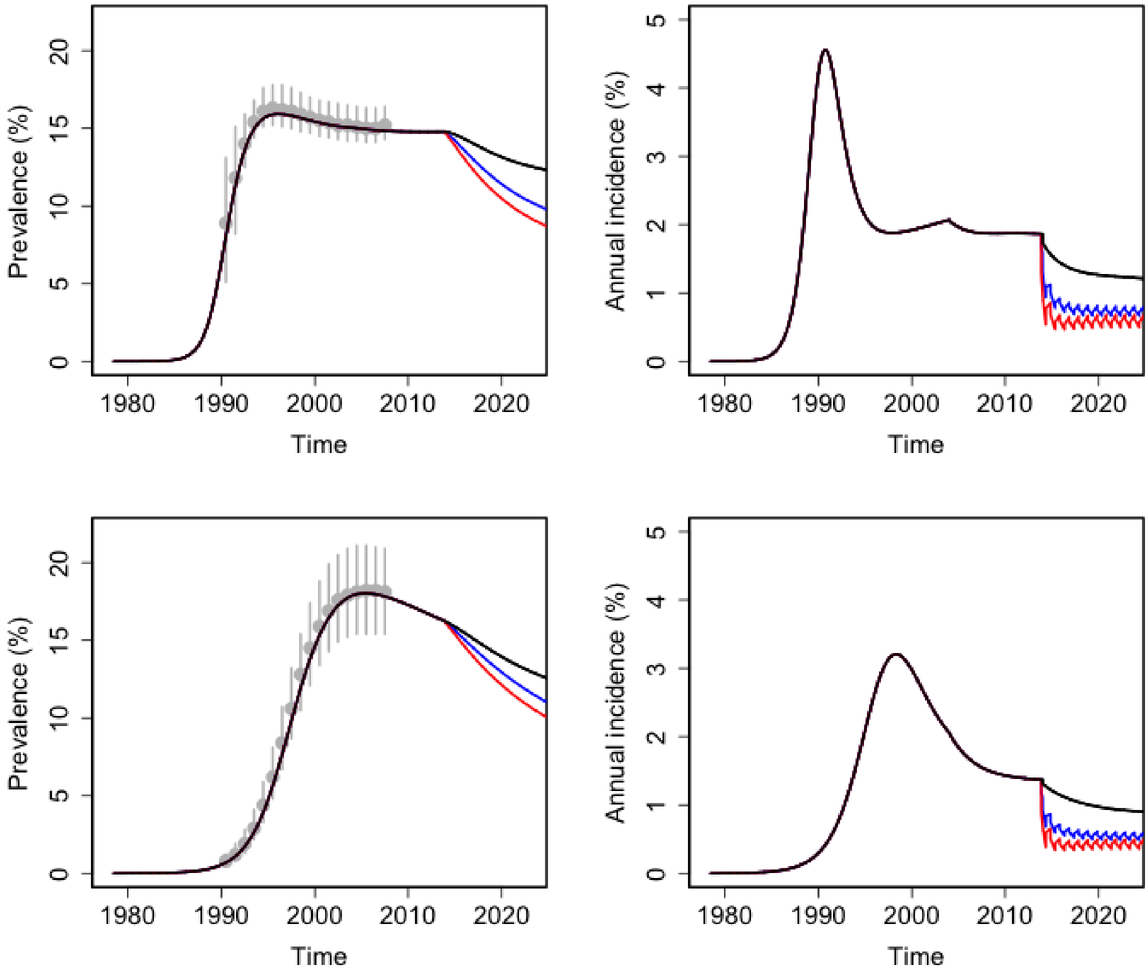

Figure 7-12: Uncertainty on the trial outcome in Zambia (top panels) and South Africa (bottom panels). The red and blue histograms show the relative reduction in 3-year cumulative incidence in arms A and B respectively when parameters vary within ranges shown in the attached manuscript (Table 2). The left panels show results obtained when all parameters are varied, and the right panels when assuming no population-level behavioural changes associated with the intervention.

Figure 7: Original paper (no change in guidelines, trial starts 1st July 2013)

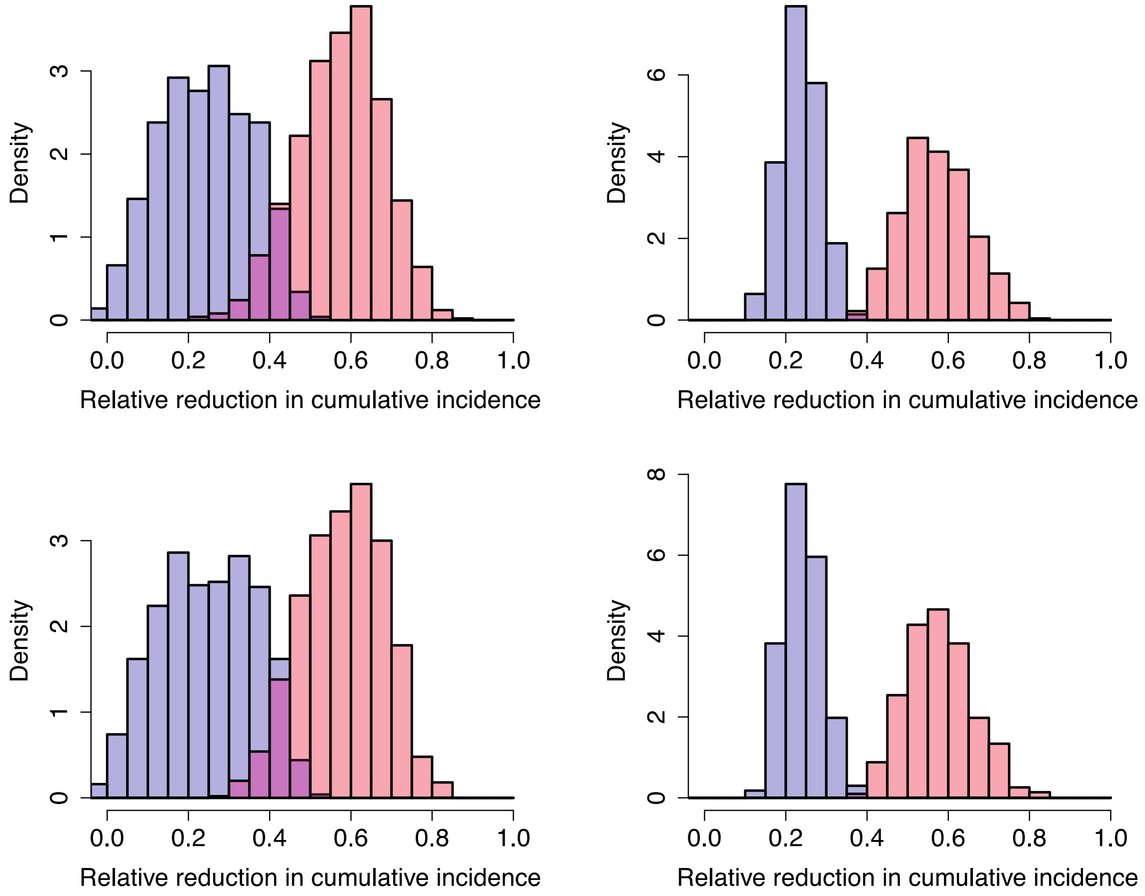

Figure 8: No change in guidelines, trial starts on 1st November 2013

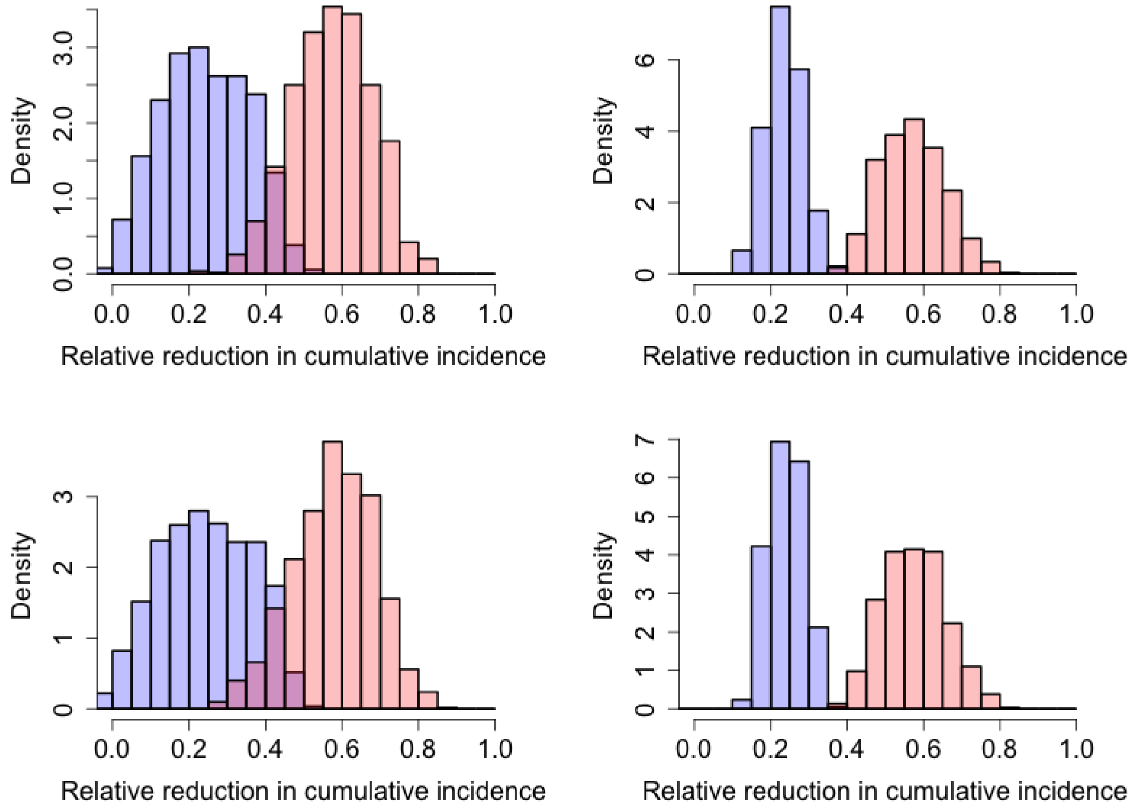

Figure 9: Late adoption of guidelines, small eligibility (see main text)

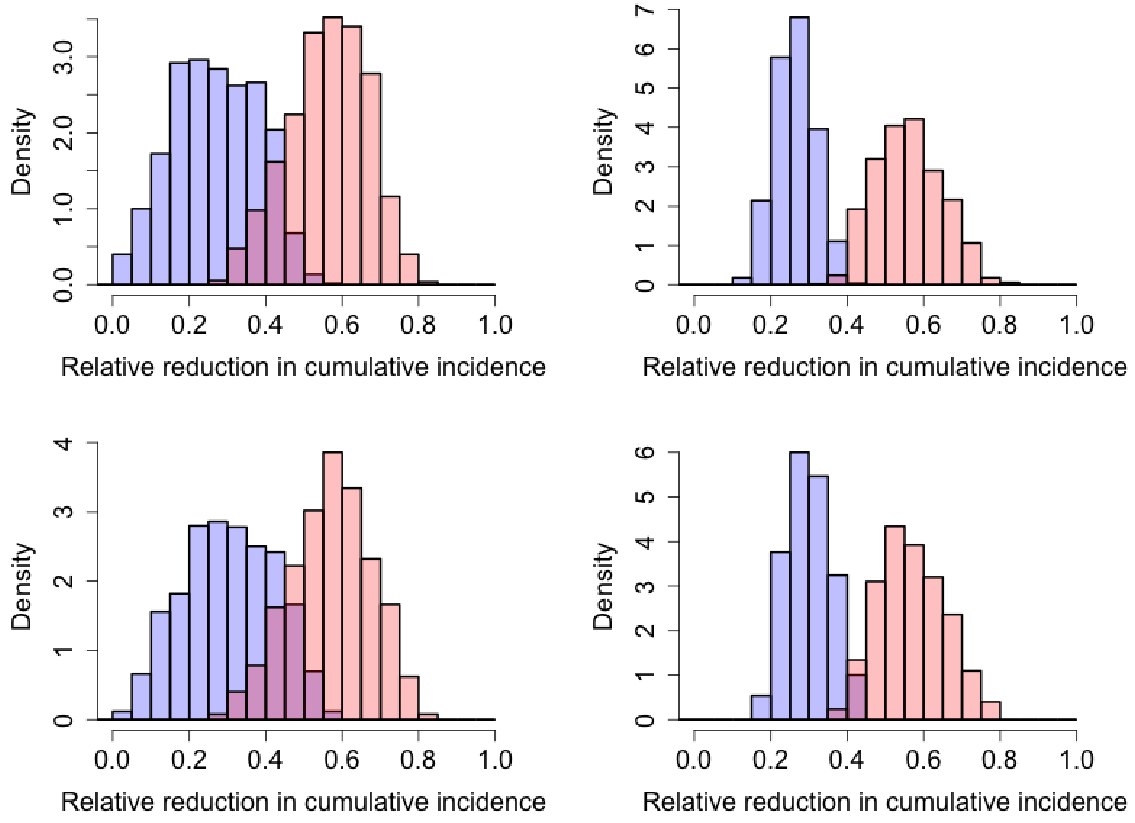

Figure 10: Late adoption of guidelines, large eligibility (see main text)

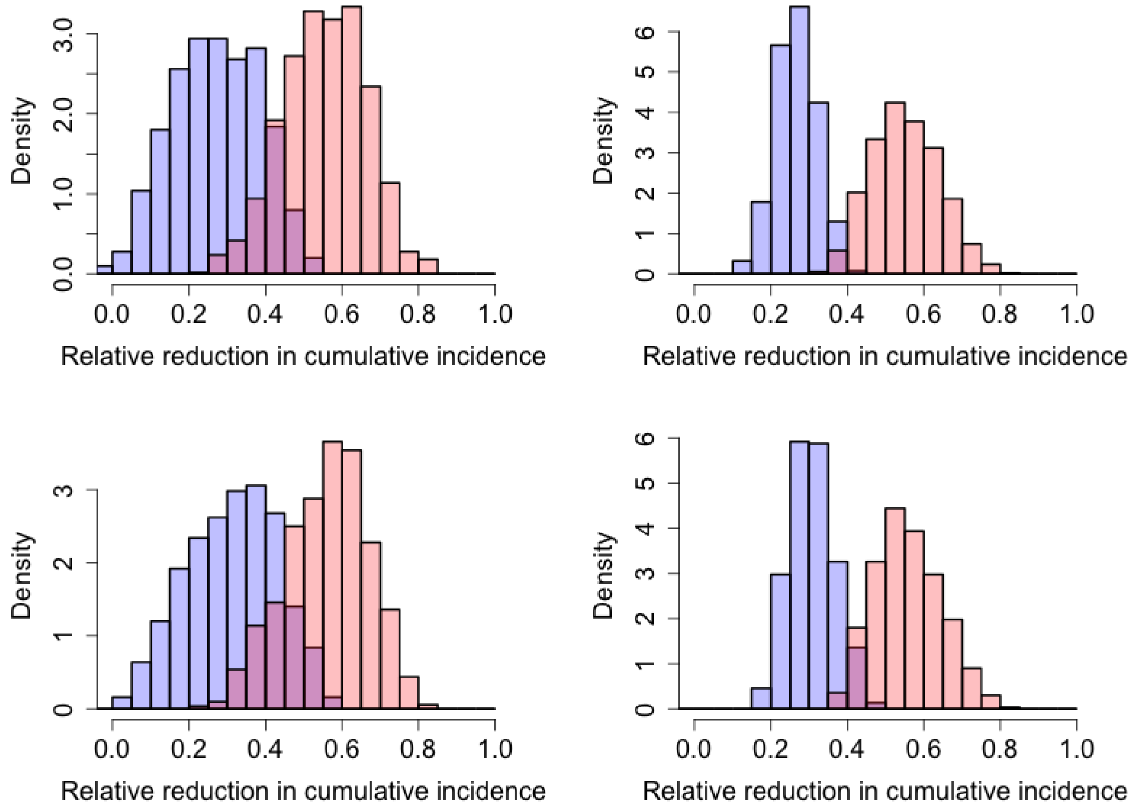

Figure 11: Early adoption of guidelines, small eligibility (see main text)

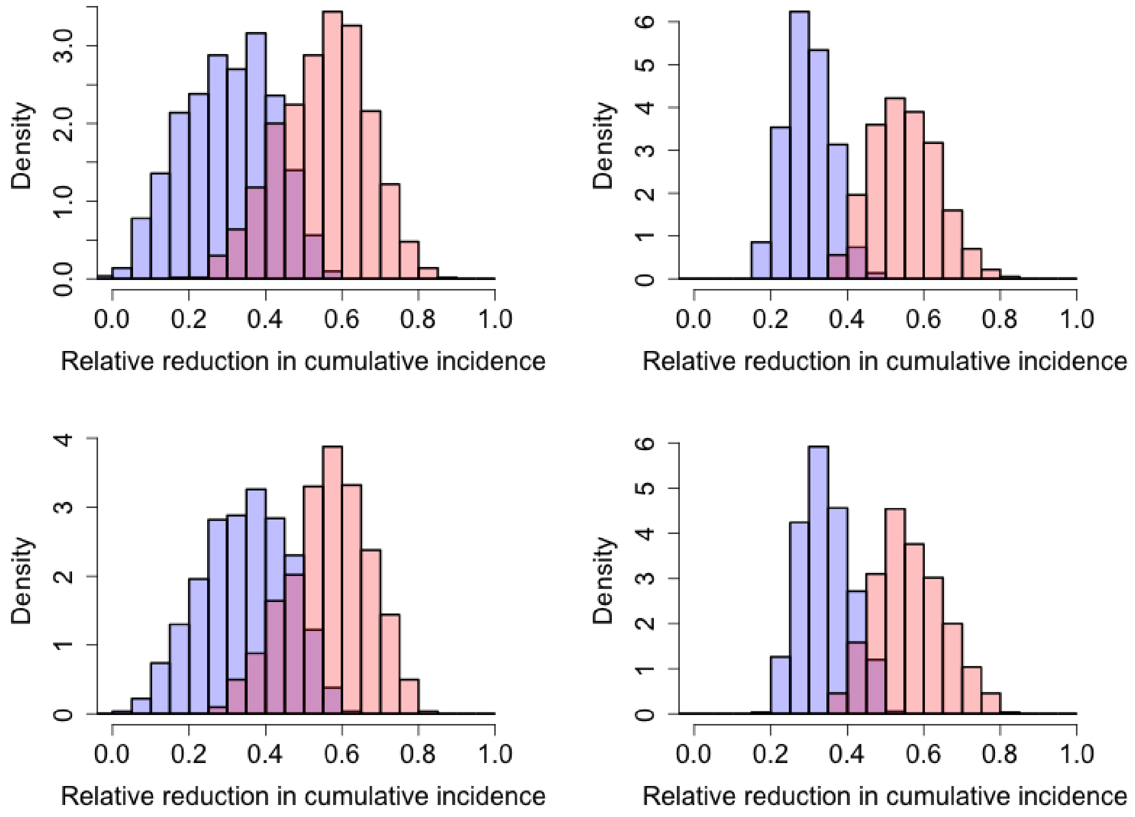

Figure 12: Early adoption of guidelines, large eligibility (see main text)

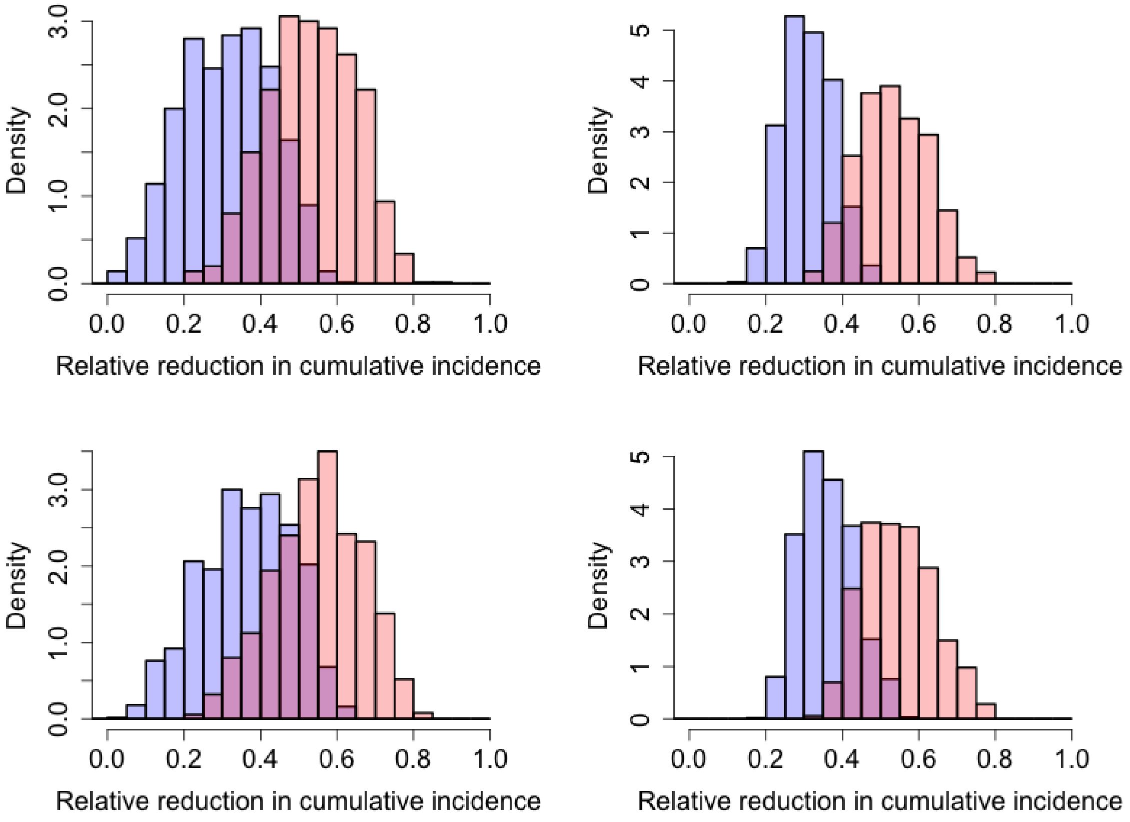

Figures 13-18: Projected impact of the intervention on HIV incidence in Arms A and B compared with Arm C for central target scenario in Zambia (top row) and South Africa (bottom row), under 10 parameter sets calibrated to the UNAIDS prevalence estimates. The red dots show the best fit parameter set.

Figure 13: Original paper (no change in guidelines, trial starts 1st July 2013)

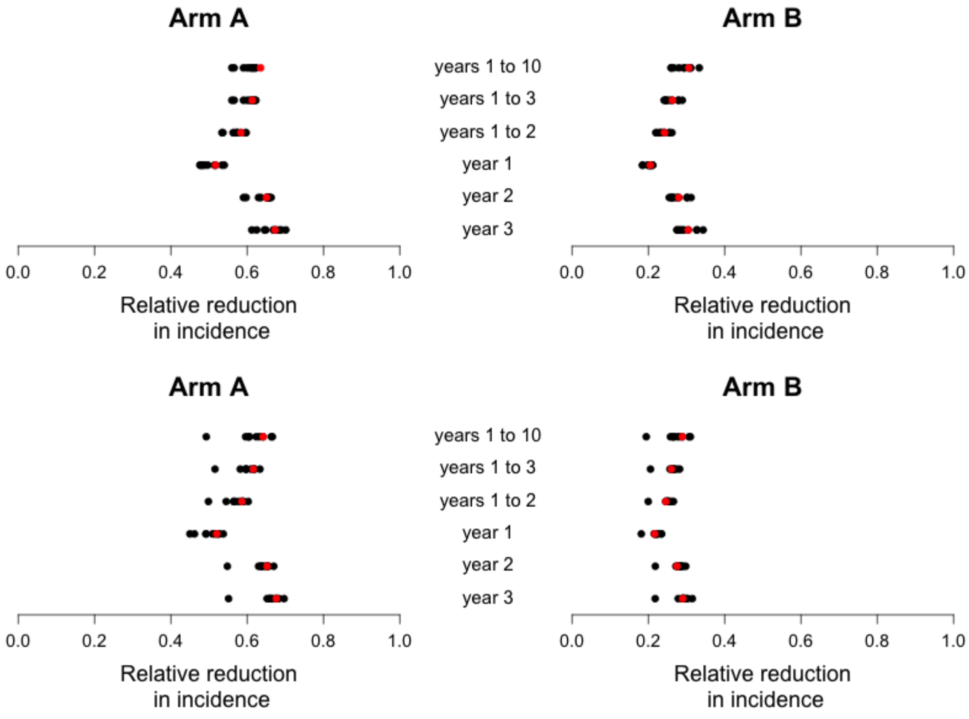

Figure 14: No change in guidelines, trial starts on 1st November 2013

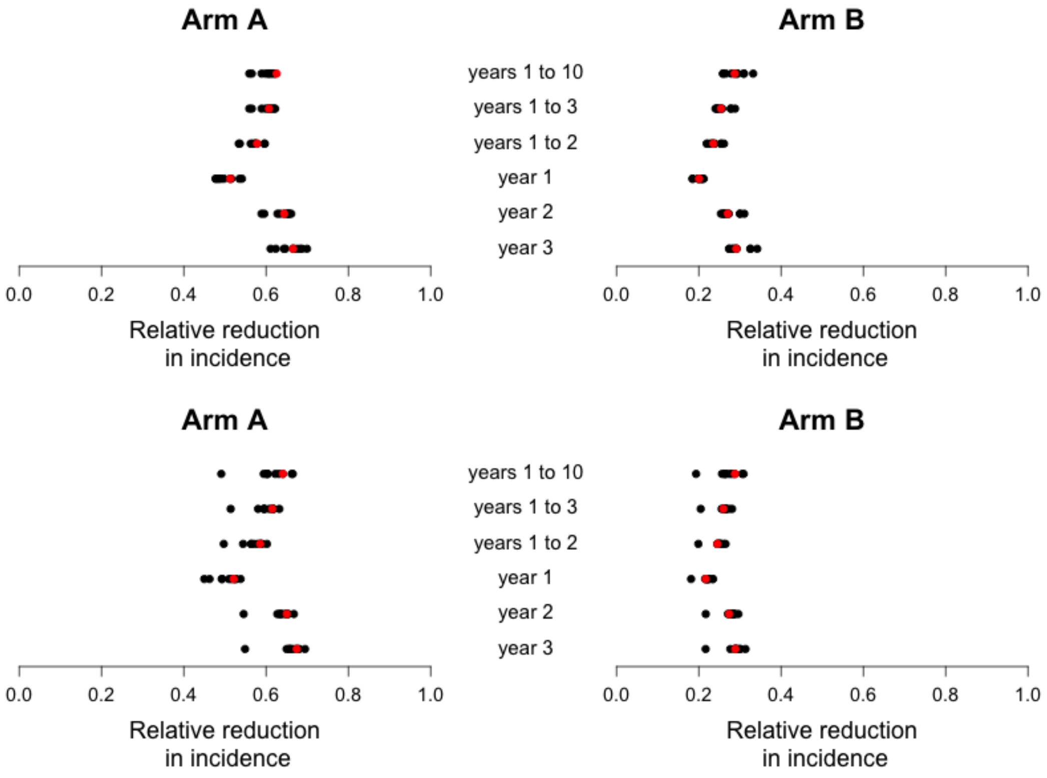

Figure 15: Late adoption of guidelines, small eligibility (see main text)

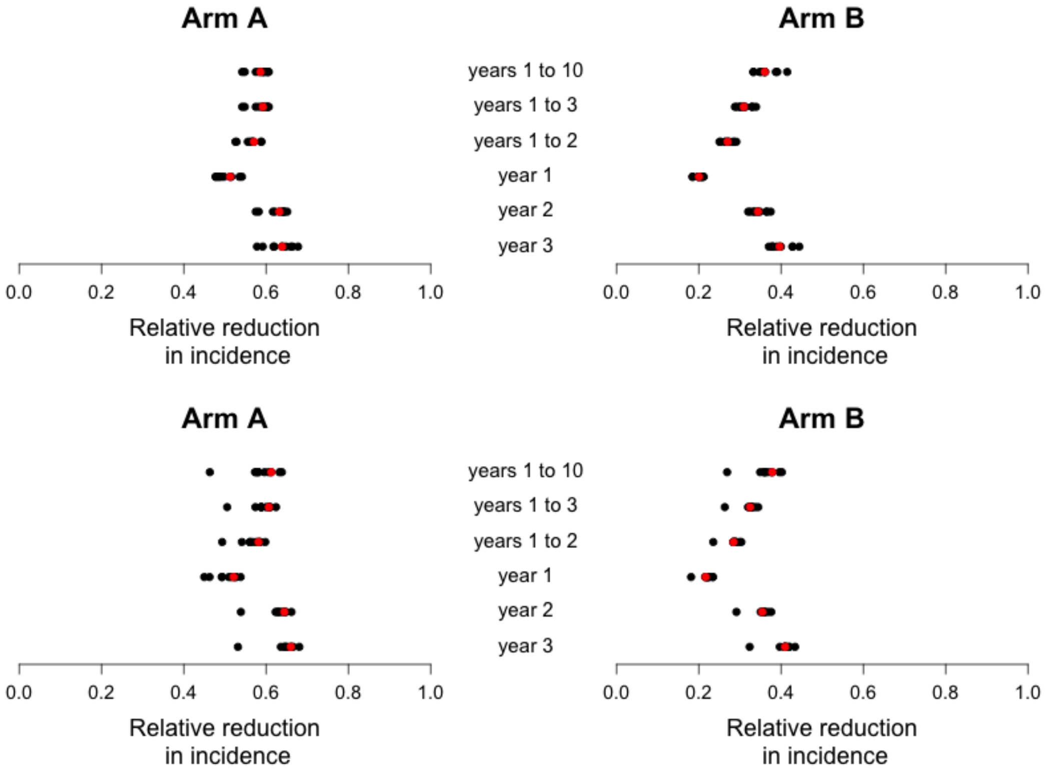

Figure 16: Late adoption of guidelines, large eligibility (see main text)

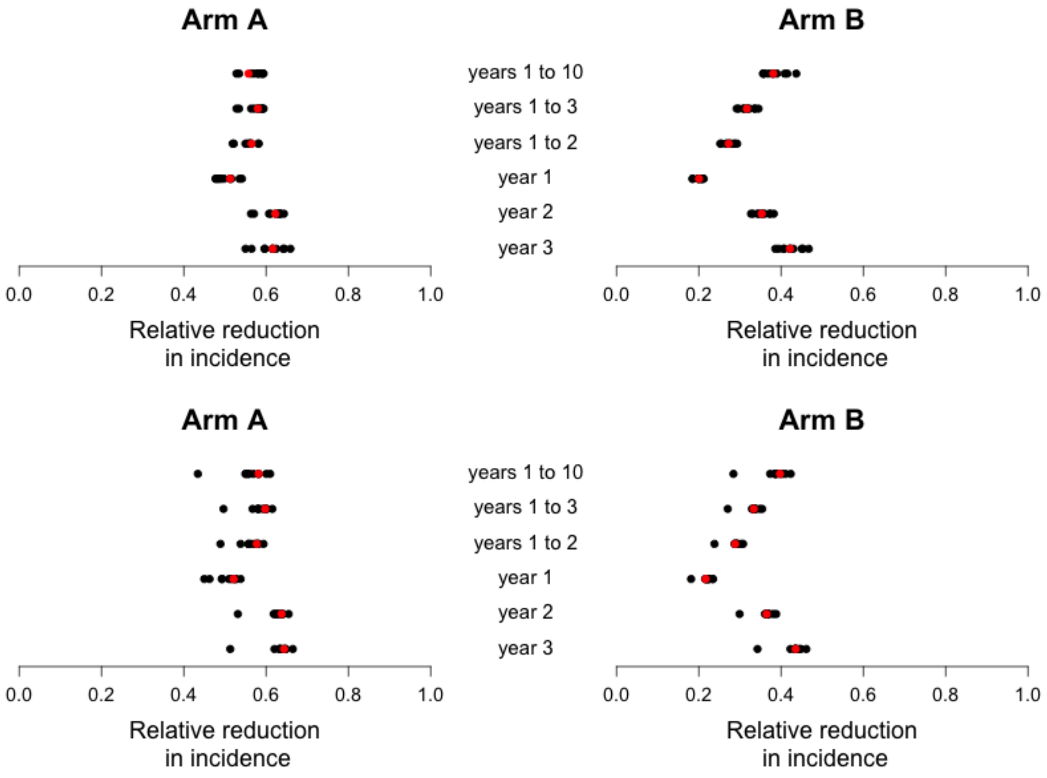

Figure 17: Early adoption of guidelines, small eligibility (see main text)

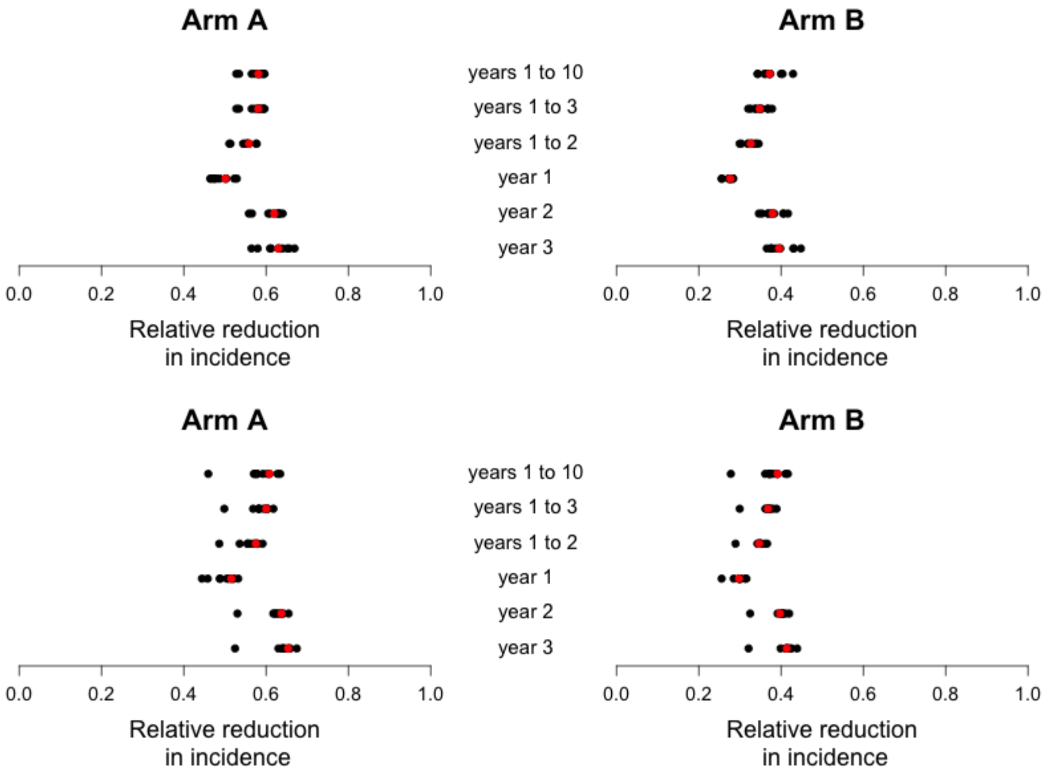

Figure 18: Early adoption of guidelines, large eligibility (see main text)

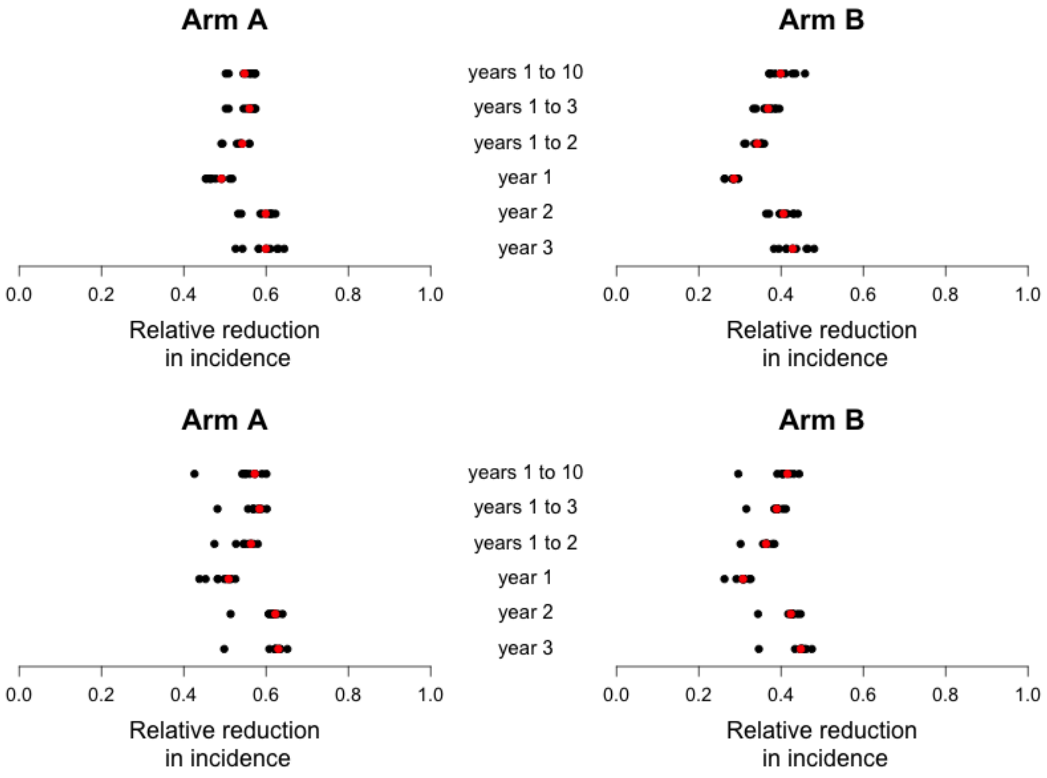

Discussion

We used a modified model to project the impact on the HPTN-071 (popART) trial of the adoption of the new WHO guidelines on ART in Zambia and South Africa.

This model does not account for individuals in pre-ART care who already know their HIV status and could initiate treatment very soon after adoption of the new guidelines, and therefore tends to underestimate the number of individuals who will initiate treatment just after adoption of the new guidelines (this effect could be studied in a future version of the model).

The model also does not account for possible disruptions in the logistics of the health care facilities associated with the increase in number of eligible individuals, which tends to counterbalance the former effect.

The anticipated date of guidelines adoption in country as well as the assumptions we have made about serodiscordant couples, co-infected individuals and pregnant women have a large impact on the predicted relative reduction in incidence between arms, however not as large as the impact of the uncertainty in parameters related to the intervention uptake and the potential behavioral changes at the community level.

Our initial model was designed following the 2010 WHO guidelines for ART initiation. The extended model we presented here assumed that the national guidelines would abruptly change from these 2010 WHO guidelines to the 2013 ones. In practice, intermediate guidelines might be implemented in-between, for instance the option B+ which has already been adopted in Zambia and South Africa, although not yet implemented. Although our model does not incorporate such features, the scenarios we have explored cover the most extreme assumptions regarding adoption of the new WHO guidelines, and hence the range of power calculations we present should cover these intermediate scenarios.

Finally, we have assumed that the trial would start on the 1st of November 2013. Sensitivity analyses (not presented here) showed that, as expected, the sooner the trial starts, the closer predictions are to our initial ones.

Appendix 1: description of the extended model incorporating adoption of new WHO guidelines in country

In this document we only describe modifications to the model.

We assume that each country adopts the new guidelines at a date t_{adopt}. Before that date, the model is the same as the initial model [2], but with an updated date of start for the trial, assumed to be the 1st November 2013

- **HIV+ adults with CD4<500 cells/mm3**

After the date t_{adopt}, we model, in all arms, an additional rate of testing and linking to care for individuals with CD4 350-500, which is equal to the background rate of testing and linking to care of individuals with CD4 200-350: with the notations of [2], $t^{2}_{test+,background}(t) = \mathbf{1}_{\{t>t_{adopt}\}} t^{3}_{test+,background}(t)$. This reflects the fact that individuals with CD4 350-500 were previously not eligible for ART initiation but become so when the countries adopt the new WHO guidelines.

- **HIV+ individuals in a serodiscordant couple or with active TB / hepatitis B**

Our model does not explicitly represent HIV+ individuals in a serodiscordant couple or coinfected with hepatitis B or active TB (SD-coinfected). If these individuals have CD4<500, they are already modeled as eligible for treatment, but not if they have CD4>500. To account for those, we assumed that HIV+ individuals in a serodiscordant couple or coinfected with hepatitis B or active TB represented a proportion $p_{SD-coinf}$ of all the HIV+ individuals with CD4>500. Moreover, we assumed that they get tested and linked to care at the same rate as HIV+ individuals with CD4 200-500, i.e. $t^{SD-coinf}_{test+,background}(t) = t^{2}_{test+,background}(t) = \mathbf{1}_{\{t>t_{adopt}\}} t^{3}_{test+,background}(t)$. At total, we approximated the testing and linkage to care of SD-coinfected individuals by an additional rate of testing and linkage to care $t^{0}_{test+,background}(t) = p_{SD-coinf} t^{SD-coinf}_{test+,background}(t) = \mathbf{1}_{\{t>t_{adopt}\}} p_{SD-coinf} t^{3}_{test+,background}(t)$ applied to all HIV+ individuals with CD4>500. This approximation should be reliable for short-term predictions, and therefore suitable for predicting trial outcomes over 3 years. The proportion of HIV+ individuals with CD4>500 who are in a serodiscordant couple or have active TB or hepatitis B is difficult to estimate. Guided by data on serodiscordant couples in several sub-Saharan African countries [3], we have assumed two scenarios, where $p_{SD-coinf} = 0.05$ ("small eligibility scenario") and $p_{SD-coinf} = 0.30$ ("large eligibility scenario"), where respectively 5 and 30% of the HIV+ individuals with CD4>500 are eligible as being SD-coinfected.

- **HIV+ pregnant and breastfeeding women**

Similarly, HIV+ pregnant or breastfeeding women (PBw) are not explicitly represented in our model. If they have CD4<500, they are already modeled as eligible for treatment. To account for those who have CD4>500, we assumed that adult HIV+ women get pregnant at a rate $w_{PBw}(t) = -\log\left(1 - \frac{b_0(t)}{0.5k}\right)$, where $b_0(t)$ is the annual per-capita birth rate, k is the proportion of adults in the population, 0.5 is roughly the proportion of women in the

population, and hence $\frac{b_0(t)}{0.5k}$ is the annual per adult woman birth rate (see [2] for sources for $b_0(t)$ and k). We further assumed that the uptake of HIV testing at antenatal clinics and subsequent linkage to care for these women is p_{test}^{PBw} . At total, we approximated the testing and linkage to care of pregnant and breastfeeding women by an additional rate of testing and linkage to care $\tau_{test+,background}^{PBw}(t) = -\mathbf{1}_{\{t>t_{adopt}\}} \log\left(1 - \frac{b_0(t)}{0.5k}\right) p_{test}^{PBw}$ applied to all HIV+ women with CD4>500. This approximation should be very reliable for short-term predictions, and therefore suitable for predicting trial outcomes over 3 years.

The uptake of HIV testing in antenatal clinics in sub-Saharan Africa is very heterogeneous, and rapidly improving [4]. Guided by data on several sub-Saharan African countries [4], we have assumed two scenarios, where $p_{test}^{PBw} = 0.40$ ("small eligibility scenario") and $p_{test}^{PBw} = 0.90$ ("large eligibility scenario"), where respectively 40 and 90% of pregnant women attend antenatal clinic and undertake HIV testing and linkage to care.

- **HIV+ children under 5 years of age**

Our model does not incorporate children so we do not model this component of the new guideline.

- **Date of adoption of the new WHO guidelines in country**

We assumed two main scenarios, where the countries would adopt the new WHO guidelines on 1st January 2014 (early adoption) or 1st January 2015 (late adoption).

- **Uncertainty analysis**

On top of uncertainty regarding the date of adoption of the new WHO guidelines in countries, as well as regarding the number of individuals affected by these new guidelines, several other sources of uncertainty might affect the predicted HIV incidence in each arm over the 3 years of the trial. First, as demonstrated in [2], several sets of parameters give relatively good fits to pre-trial estimates of national HIV prevalence by UNAIDS. Second, there is large uncertainty on what will be the uptake of the PopART intervention in arms A and B.

To explore the extent to which those sources of uncertainty influence the power of the trial under the new WHO guidelines, we ran the model under several parameter sets that fitted the pre-trial national HIV prevalence estimates by UNAIDS (see [2] for the parameter sets used). Moreover, for the best fitting parameter set, we explored a range of values for parameters relating to the intervention uptake (see [2] for more detail).

Appendix 2: power calculations

Power calculations were performed as described in the HPTN071-PopART protocol. Table 3 below presents additional power calculations for scenarios where the difference in HIV incidence between arms A and B is less marked (see Table 7 in protocol for other scenarios).

Table 3: Power for comparison of HIV incidence between Arms A and B, with 7 communities per arm and Population Cohort of 2500 adults per community (assuming that on average 2125 (85%) will be HIV-uninfected at baseline and that loss to follow-up will be 20% after 2 years and 25% after 3 years)

HIV incidence rate/ 100py (control arm)	Between-cluster coefficient of variation (k)	Effectiveness (%) Arm A	Effectiveness (%) Arm B	Power (%)
1.00%	0.15	55%	35%	66%
1.00%	0.15	55%	40%	46%
1.00%	0.15	60%	35%	87%
1.00%	0.15	60%	40%	72%
1.00%	0.2	55%	35%	55%
1.00%	0.2	55%	40%	37%
1.00%	0.2	60%	35%	76%
1.00%	0.2	60%	40%	61%
1.50%	0.15	55%	35%	76%
1.50%	0.15	55%	40%	55%
1.50%	0.15	60%	35%	93%
1.50%	0.15	60%	40%	82%
1.50%	0.2	55%	35%	62%
1.50%	0.2	55%	40%	42%
1.50%	0.2	60%	35%	83%
1.50%	0.2	60%	40%	68%

References

1. World Health Organization (2013) Consolidated guidelines on the use of antiretroviral drugs for treating and preventing HIV infection.
2. Cori A, Ayles H, Beyers N, Schaap A, Floyd S, et al. (2013) HPTN 071 (PopART): a cluster-randomized trial of the population impact of an HIV combination prevention intervention including universal testing and treatment. 2. Mathematical model. Submitted.
3. Chemaitelly H, Cremin I, Shelton J, Hallett TB, Abu-Raddad LJ (2012) Distinct HIV discordancy patterns by epidemic size in stable sexual partnerships in sub-Saharan Africa. Sex Transm Infect 88: 51-57.
4. USAID (2013) Demographic patterns of HIV testing uptake in sub-Saharan Africa.

The following is a copy of the manuscript submitted for publication to PLoS One, which describes the initial model developed to assist the HPTN071-PopART trial.
